# Collaborative and Reproducible Research: Goals, Challenges, and Strategies

**DOI:** 10.1007/s10278-017-0043-x

**Published:** 2018-02-23

**Authors:** Steve G. Langer, George Shih, Paul Nagy, Bennet A. Landman

**Affiliations:** 1 Radiology, Mayo Clinic, Rochester, MN USA; 2 Department of Radiology, Weill Cornell Medicine, New York, NY USA; 3 Russell H. Morgan Department of Radiology and Radiological Sciences, Johns Hopkins University, Baltimore, MD USA; 4 Electrical Engineering, Vanderbilt University, Nashville, TN 37235 USA

**Keywords:** Machine learning, Computers in medicine, Computer analytics

## Abstract

Combining imaging biomarkers with genomic and clinical phenotype data is the foundation of precision medicine research efforts. Yet, biomedical imaging research requires unique infrastructure compared with principally text-driven clinical electronic medical record (EMR) data. The issues are related to the binary nature of the file format and transport mechanism for medical images as well as the post-processing image segmentation and registration needed to combine anatomical and physiological imaging data sources. The SiiM Machine Learning Committee was formed to analyze the gaps and challenges surrounding research into machine learning in medical imaging and to find ways to mitigate these issues. At the 2017 annual meeting, a whiteboard session was held to rank the most pressing issues and develop strategies to meet them. The results, and further reflections, are summarized in this paper.

## Introduction

The mission of the SiiM Machine Learning Committee (SiiM-MLC) is to educate, promote, and advance the state of the art in medical imaging research. To be sure, there are many issues to be addressed in the arena of machine learning (ML) applications in medical imaging, and many of them are not unique to the field. For example, anyone performing medical research on humans in the US must be:

1. in compliance with HIPAA, HITECH, and institutional review board (IRB) requirements [1]
2. capable of defining inclusion criteria in alignment with hypothesis-driven research
3. capable of locating relevant data sets to perform research on
4. able to design, execute, and report reproducible results of experiments
5. publish articles with data and analytics in a reproducible and unbiased manner.

The members of the SiiM-MLC have well over a century of combined experience in the technical issues surrounding medical imaging research, but are also well aware that in addition to the research concerns, new challenges arise when translation from lab to bedside is contemplated. Legal and ethical questions must be asked and answered which often requires education of lawmakers. Lawmakers, in turn, rely on the regulatory agencies to ensure that the new technology is deployed in safe and efficacious forms. Finally, non-experts in the field must be educated and licensed in the safe use of the new tools on patients.

While ML is very data hungry and requires new tools operating at new scales, protected health information (PHI) requirements continue to get more stringent. Given these challenges, some of the authors met with other practitioners at the Research Whiteboard session at SiiM 2017 to chart a course through the ML minefield from novice to expert and identify key obstacles. It was made abundantly clear that while the tools used may differ, the desires are nearly the same.

The remainder of this work sets forth an exposition of technical challenges to be addressed and mitigations to meet them. To put some structure on this discussion, we will break these areas into goals, challenges, and strategies.

## Goals

To appreciate the issues that complicate research, it is helpful to contrast it with the normal clinical workflow. Clinical workflow is driven by medical need, that is, the clinician does not have a choice of what cases will arrive for care. Rather, the scenario is event driven on a first come, first served (subject to triage) sequence of uncorrelated cases. The resulting radiology clinical workflow (simplified) looks like this:

- Radiology clinical workflow

1. Patient arrives and is registered at medical center
2. A clinician orders a new exam for a patient
3. The interpreting radiologist reviews the order and determines the relevant protocol to address the clinical question
4. The night before patient arrival (or immediately if ad hoc), relevant compares are fetched for the patient from vendor neutral archive (VNA) to picture archiving and communication system (PACS)
5. New exam is acquired
6. Post-processing and/or QA occurs
7. Priors and QA’ed new exam are sent to radiologist on PACS for interpretation
8. Radiologists read new studies in comparison to priors, makes measurements, and reports case
9. Reported exam is archived to VNA

Now, in contrast with the above, the steps and workflow required for retrospective research are different. The researcher wants to find patients or exams that are relevant to their question, have low barriers (in money and person hours) to access data needed to start their research, have the means to duplicate their results, and ideally have the option to collaborate with others. For example, a researcher may be trialing a new therapy in which case they need to find patients with that disease. Another researcher may want to find a training set for a new diagnostic machine learning algorithm aimed at diagnosing a specific disease—in which case, the search is for prior studies (labs, imaging, etc.) that have a known state (positive or negative) for those disease findings. Broadly, the workflow steps look like this:

- Research workflow (retrospective)

1. Define the scope of the issue to be studied (i.e., inclusion criteria)
2. Perform cohort discovery (against patient or exam results databases as appropriate for inclusion criteria)
  - Often by a research administrator acting as an honest broker who is allowed to see PHI
  - Grouping of imaging studies temporally (i.e., lesion tracking) or combining multi-modalities
  - De-identify the cohort members (ideally done prior to the being seen by the principal investigator (PI) (i.e., blinded with only the research administrator having the PHI-de-identified key)). This point is essential if incidentalomas are discovered during the trial and the PI needs to report the finding back to the patient’s care team
3. Assemble the de-identified cohort and perform randomized trials
  - If a therapy trial, on the patients
  - If a diagnostic trial, on the new analytic and runtime platform
4. Perform measurements and analysis
5. Record results to another database
6. As a bonus, be able to compare results with others and/or collaborate on algorithm improvements

As many have commented (and as was shared by the whiteboard attendees), points 2–3 tend to be the first large barriers to overcome for sites beginning a research program, particularly, since such searches tend to be on free text and not machine-discoverable [2]. It may also be the case that image studies are scattered across numerous archives, requiring multiple searches [3]. Even for single-site institutions, the tools generally available are not flexible enough to perform these queries and other related tasks (e.g., data aggregation). In particular, a given site may have to span queries across multiple systems with multiple patient and exam identifiers in order to perform cohort identification. Also, everyone struggles with de-identification and it was acknowledged as an ongoing issue. So a simplifying question was asked for those sites just beginning their research programs, “Do you require access to your own site’s data or if a sufficiently rich and curated public dataset (meta-tagged to enhance cohort discovery) were available, would that be an acceptable alternative?”

In many cases, it turns out the answer to the above question was “yes.”

Among sites with established research programs, the next pain points were related to the costs and complexity of maintaining an array of IT systems of various types to host different analysis tools. For example, some analytics can scale across *compute* clusters and benefit from something like LUA pipelines or grid engines [4, 5]. Other sites more involved with finding patterns in textual data (e.g., big data) require database clusters that scale across database nodes [6]. And of course, practitioners looking for patterns in image data require specialized ML knowledge in software “stacks” that layer ML learning engines with python or R (for analysis) and graphics processing unit (GPU) libraries for runtime [7–9].

Finally, the workflow inherent in bringing a new analytic to clinical practice needs addressing, i.e., the translational problem. There are really two steps in this; first, demonstrating the new tool is efficacious during a proof-of-concept pilot, then (assuming success) expanding the pilot to normal clinical workflow.

- Translation to practice workflow (prospective)

1. Patient is scheduled for an imaging study.
2. At protocoling time, case is reviewed for possible inclusion in the IRB project
3. If suitable, patient is consented and study is “tagged” for both the standard of care workup and the new research workup.
4. In post-study acquisition, the two workups are de-identified and presented to expert observers to assess efficacy.
5. Assuming success (and legal hurdles met), the new tool is:
  - commissioned into the current standard of care armamentarium
  - and guidelines (or automated systems) are created to route candidate studies to the new tool as in the normal clinical workflow and
  - patients benefit.

## Challenges

The following issues were identified in the whiteboard sessions as impediments to productive research in diagnostic imaging analytics. A casual inspection reveals that the majority of issues (four of five) are related to the availability of relevant, de-identified, and curated datasets.

(I) Cohort discovery

- Insufficient or inaccurate meta-tagging to comprehensively locate all potential participants that match the inclusion criteria:
  - Inclusion criteria span multiple systems (labs, pathology, radiology, surgical notes)
  - Inclusion criteria elements are unstructured (i.e., free text not amenable to SQL queries) necessitating an NLP query tool of radiology reports and/or electronic health record (EHR) data

Most clinical systems in imaging have not been designed in a way to locate all the potential participants that match inclusion criteria for a particular machine learning cohort. Inclusion criteria may be imaging-based findings (e.g., pulmonary nodules for lung cancer screening CT studies) which are mostly in radiology reports, or the criteria may be more complex (e.g., sarcoidosis with imaging and clinical features) with data in both radiology reports and in the EHR. Currently, most radiology reports and EHR notes are still free text, even though they may have structured headings, and would necessitate some sort of NLP query for it to be effective.

(II) Dataset sparsity
  - Insufficient samples to represent all the stages of a disease
  - Insufficient samples for the statistical power needed by a trial or training set

For the current DL algorithms to be effective in delivering good ‘Narrow AI’ results, they need have reasonably large number of examples of a particular disease finding—typically several hundred or several thousand samples. For many diseases, dataset sparsity can be a big issue. There may be insufficient samples to represent all the stages of a disease or insufficient samples for the statistical power needed by a trial or machine learning training set, even given different data augmentation techniques. Data sparsity may ultimately limit the kinds of machine learning problems that one can work on.

(III) De-identification
  - Assuring all PHI (including in image bitmaps) is aliased, hashed, blanked, or offset as per applicable government regulations [10]
  - While achieving the above do so without destroying (in the case of DICOM images) private tags that may be required by analytics
  - Assuring all data relating to a given real patient is mapped to the same alias
  - Liability concerns if all PHI is not scrubbed.

Like any research project, robust de-identification of any machine learning dataset is required before use. All PHI (including in images) should be aliased, hashed, blanked, or offset as per applicable government regulations, while preserving some private tags that may be required by analytics. De-identification mechanisms will be required for ensuring all data relating to a given real patient is mapped to the same alias.

(IV) Unintended reidentification of datasets

Black hats can reconstruct patient’s faces (or other features) from high-resolution CT or MR and run those facial images against public databases to identify patients. To mitigate this threat, there is so called defacing software to prevent these types of reidentification [11]. It should also be stressed that such mitigations should be pursued as close to the source systems as possible to reduce the chances of man-in-the-middle compromises [12].

(V) Building, populating, and maintaining the runtime platform
  - Servers (CPU, GPU, grid)
  - Storage
  - Orchestrating workflow steps on the runtime platform
    - Bringing the correct inputs to the runtime platform
    - Performing computations
    - Passing results to the required downstream analytics
    - Validating execution of all workflow steps
    - Storing results back to the database(s)

Delaying V for now, it is useful to address the remaining above points. Let us consider items III and IV first. There are many publicly funded open datasets [13]. While perhaps not perfectly de-identified, these resources at least remove primary liability from the individual researcher for any de-identification issues. Points I and II (data set sparsity and cohort discovery) require more discussion. Data set sparsity has several facets: a relatively acute disease that is not well represented in the archive vs. more chronic diseases whose state evolves over time. The former can be addressed with synthetic methods of data augmentation on a base dataset [14, 15]. The latter requires a systematic effort to find examples of all disease states that have significant impacts on imaging findings [16].

Cohort discovery is perhaps the most important issue to be solved. At several recent meetings, it was noted that while open archives (i.e., the Cancer Imaging Archive (TCIA)) may have many thousands of studies, it is not easy to find studies relevant for a given research project [17]. In particular, it was suggested that there are two major shortcomings in today’s open archives with respect to automated discoverability: meta-tagging with a standard ontology and an API that allows automated mining by programmable agents. {Note: While TCIA does have a RESTful API, a researcher would have to brute force search through the archive and open at least each series to search for relevant studies based on the normal DICOM tags (i.e., for anatomy, or series description). Without standard terms that encode the study findings or acquisition parameters, however, this approach would still require further processing by the researcher to determine applicability [18]}. Others have made similar observations [19]. These points will be addressed in the next section.

## Strategies

Many of the points listed in the “Challenges” section are related to the issues surrounding ML *retrospective* research. However, the final issue under the “Goals” section was translating research to proof of concept, then clinical pilot/FDA validation, and finally routine clinical care. In this section, we will develop a hierarchical cognitive model to organize the requirements/methods needed to address the challenges laid out above.

As stated in the “Introduction” section, the mission of the SiiM-MLC is threefold:

- educate
- promote and
- advance the state of the art

**Fig. 1:**
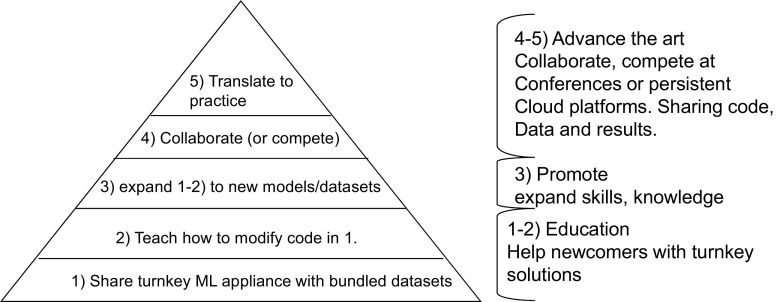
Conceptual pyramid of the objectives of the SiiM Machine Learning Committee (MLC). The committee’s github site has turnkey solutions (Dockers) to enable newcomers to the ML field get started without having to master multiple technology dependencies first (level 1–2). Then, as the person grows their knowledge, additional resources guide them to how to expand those base Dockers to address new problems (level 3). At the upper levels, the MLC aims to foster guidelines to conducting reproducible science, provide shared datasets, and foster collaborative research

These words, and how they relate to each other, are expressed in a concept pyramid in Fig. 1; education is addressed by levels 1–2 where tools are developed to share code and data *in a portable way* among students and early investigators. Promotion occurs at level 3 where new and enhanced training data sets become available to broaden the disease models developed from given software frameworks. Finally, the state-of-the-art is advanced via collaboration (competition?) among researchers using different algorithms on the *same data sets* in a collaborative environment, and ultimately translation to clinical use.

(A) Education

SiiM-MLC is addressing basic education issues in ML through the use of open source tools and open datasets where possible. A key strategy is to develop turnkey “appliances” that get novices up to speed quickly on the tools required for ML work, without mastering all the underlying technologies first. Next, novices are pointed to open curated datasets relevant to the appliance they have, to offload the de-identification chores mentioned previously. As of this writing the MLC has published:

- *A turnkey ML appliance* at the SiiM-MLC github site [20]. It bundles in a single Docker a combination of tools (e.g., Keras, Tensorflow, Jupyter) that enable running ML as an appliance. {Aside from that, for the novice, a Docker is a “container” for a bundle of software that runs as if it were its own computer [21, 22].} The availability of this Docker obviates the need for the novice to learn at least four different technology stacks just to start playing with ML. To run a Docker, one need only install the Docker runtime engine on one’s computer [23].
- *Use of existing open datasets.* Many open imaging datasets already exist (e.g., TCIA) and SiiM-MLC will promote the use of those existing datasets when possible. This approach presents minimal legal risk to SIIM and where those datasets have already been curated (e.g., prostate cancer), it significantly reduces the effort required for ML researchers to begin work.

(B) Promote

In many cases, there are demonstration data sets sufficient to exercise the ML Dockers which the SiiM-MLC has published. For these data, the Docker authors are tasked with bundling (via URL references) the recommended starter data sets with their published software on the github site. However, when a researcher wishes to extend a given ML framework to new diseases, new datasets may be required. At this point several concerns arise: are sufficiently deep datasets available, are they de-identified but still useful, and are they curated, annotated, and discoverable by a researcher? To address these needs, the MLC seeks to partner with industry and academic sites to identify new datasets, crowdsource curation and meta-tagging using existing (or to be developed) tools, and share these data set locations back on the github site [24].

It is also often the case that many ML developers are willing and eager to share their code and algorithms, but they may have developed on a platform with specific expertise required. By encouraging adherence to a common development architecture, the SiiM-MLC is aiming to reduce impediments to code sharing.

(C) Advance the state of the art

*By definition*, reproducible science requires being able to reproduce results. Without access to another researcher’s code *and* data, there is no way a third party can duplicate that researcher’s results. Github and Docker vastly lower the learning curve required to share *code and runtime environments—*for those who want to. What they do not address is the commonality of *datasets*.

By providing a collection of respected runtime appliances at the SiiM-MLC github site, and recommended datasets that are appropriate for them, much of the learning curve is reduced for all but researchers at the bleeding edge of ML investigations. To address the needs of *that group*, the MLC is working in conjunction with the Conference on Machine Intelligence in Medical Imaging (C-MIMI) and the SiiM Hackathon committee. Briefly, the current Hackathon site supports a fast healthcare interoperability resource (FHIR)-based EMR server, and a DICOM web-based VNA [25]. Together, these servers expose five patient personas of correlated data sets that enable students to develop new applications with realistic data.

The reader will recall from the last paragraph in the “Challenges” section the two major points regarding cohort discoverability; the first is the lack of a common ontology to meta-tag studies with and the second is the lack of machine “mineable” interfaces to simplify the tasks of finding relevant studies for a given use. At C-MIMI 17, there was great interest among attendees to partner with other agencies to enhance both these areas. As a result, two new members have joined the MLC (one from NCI, the other is the chair of the RADLEX Committee). In cooperation with the SiiM Hackathon committee, there is a proof of concept effort underway to demonstrate:

- SiiM-sponsored MLC ontology on “live” data
- crowdsourced meta-tagging of their dataset
- FHIR-based mining on the ontology

**Fig. 2:**
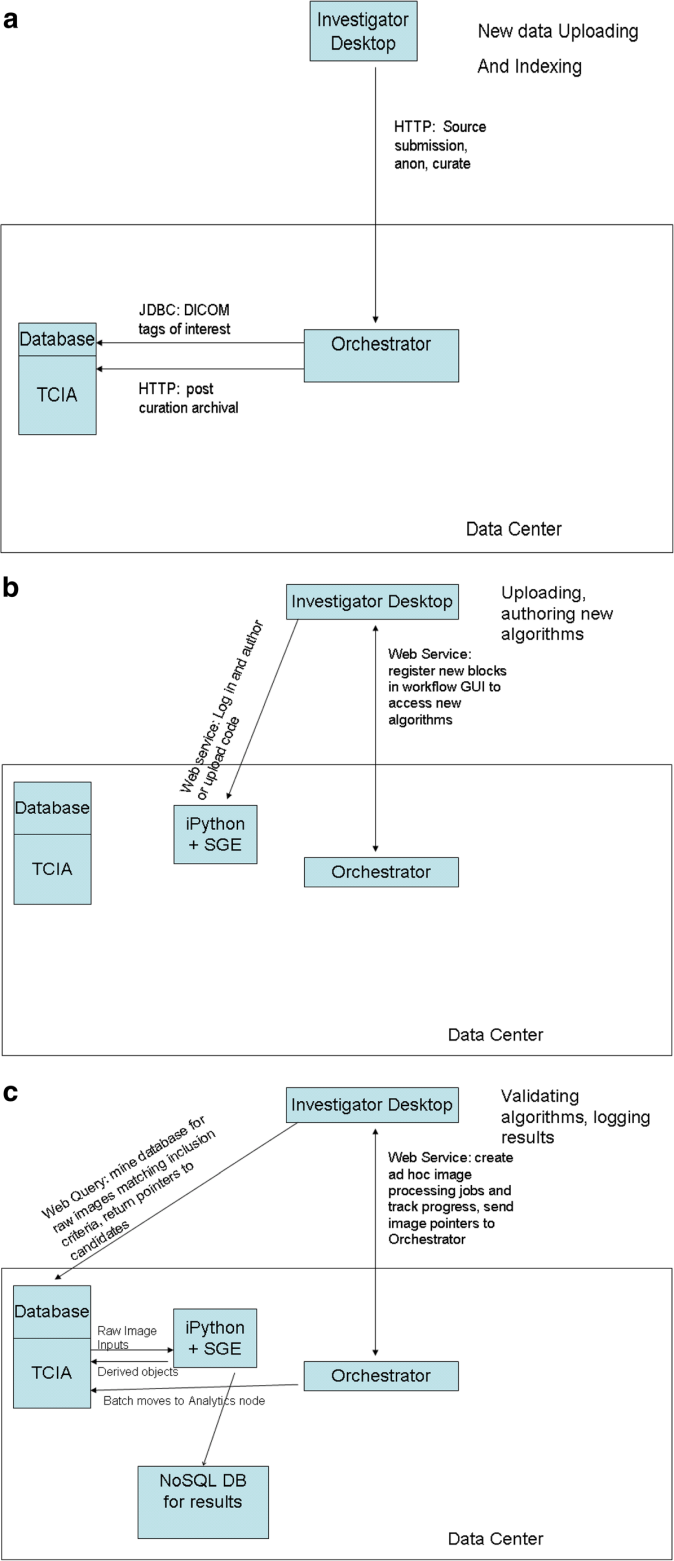
Functional requirements for a cloud-based platform for conducting reproducible ML research. **a** Users upload de-identified data; the Orchestrator checks the submission in, holds it for curation, and crowdsources meta-tagging. **b** Another investigator uploads their model to try it out on the runtime platform on the existing datasets. The Orchestrator checks the code in and assigns access rights as per the author’s wishes. **c** The investigator locates data relevant to their project, submits it to their algorithm, and stores results back to the system. For greater detail, see the text

The foregoing addresses many of the challenges. However, one point has been deferred until now and that is the many points that need to be considered to address the issues in “Challenges” section V.c in a scalable, collaborative, and secure manner. One way to address these needs is a cloud-based collaborative environment (as shown in Fig. 2) where image sets, analytics, and runtime platforms are co-located. The system indexes code and data, enforces access controls, and collects runtime results in a single location—obviating the need to every investigator to pull image sets to their own locations. Ideally, such a cloud platform should consider the three key uses cases below and deliver on their requirements:

(A) Uploading new image sets: They need to be de-identified, curated, tagged for discovery, and optionally tagged for restricted access. To support these requirements, both users and uploaded data have to belong to one or more groups. Access is regulated to assure a user can only see images if they are the owner, in the same group as the image owner, or the image set is tagged as public. The Orchestrator performs many roles. First, it assures images are HIPAA compliant. Then, it alerts curators there are new images to curate. After curation (and review by a second curator), applied meta-tags are stored to the database to enable discovery. In this example, the site is leveraging the TCIA archive.
(B) Uploading new algorithms: Similar to the above, users publish their algorithms (possibly in Docker form) to the site or develop on the site’s cloud-based development platform. As before, the code is tagged by the owner with access controls (publicly viewable/usable—or private—usable only by members of the same group). The Orchestrator checks the new code into the site, making it available as building blocks in workflows for authorized users
(C) Executing ML experiments: This is where the aforementioned elements come together. Using a web-based user interface, users select the image set of interest (assuming access controls allow it) as an input to a workflow consisting of one or more analytics. The UI allows users to manipulate the registered code blocks, collect results, and publish them back to the site’s database (relational or not). A simple example could be an investigator who wishes to try to develop an ML model for finding emphysema, steps are:
  - select low-dose thoracic CT studies that are known to have occurrences for the findings of interest
  - send them to a CT denoising algorithm, then route output to
  - a segmentation engine to subtract out the lungs, send the result to
  - a deep learning model that annotates positive cases, then
  - compare the selected positive cases to the known results in the database and record the results
  - repeat the above for different ML models to evaluate

To deliver the functions outlined above, there are numerous implied capabilities:

- capable de-identification algorithms
- crowdsourcing tools that interfaces with the Orchestrator to enable the work of human (or AI based) annotators to meta-tag data
- a well-defined API for investigators to query and find relevant studies for their algorithm
- well-defined APIs to fetch studies to the runtime environment, and pass outputs from one processing block to the next in the workflows hosted by the Orchestrator

## Discussion and Conclusions

The members of the SiiM-MLC are dedicated to enumerating and mitigating (where possible) the challenges in the field. For new investigators, the learning curve of starting up a runtime platform and locating initial image sets has been significantly reduced by the MLC github site.

For intermediate researchers wishing to help advance the field, the MLC is partnering with open archive sponsors and ontology experts to drive automatable cohort discovery. The resulting tooling and ontology standards will be shared back to the community for broader use and demonstrated on existing open archives.

Finally, for those who wish to participate in open challenges, the MLC is investigating sponsors interested in developing cloud platforms along the architecture outlined in Fig. 2. Several industry partners have already shown interest in joining the MLC to host datasets and runtime platforms.
